# EzBioCloud: a genome-driven database and platform for microbiome identification and discovery

**DOI:** 10.1099/ijsem.0.006421

**Published:** 2024-06-18

**Authors:** Mauricio Chalita, Yeong Ouk Kim, Sein Park, Hyun-Seok Oh, Jae Hyoung Cho, Jeongsup Moon, Nuga Baek, Changsik Moon, Kihyun Lee, Junwon Yang, Gi Gyun Nam, Yeonjae Jung, Seong-In Na, Michael James Bailey, Jongsik Chun

**Affiliations:** 1 CJ Bioscience Inc, Seoul, 04527, Republic of Korea; 2 Interdisciplinary Program in Bioinformatics, Seoul National University, Seoul, 08826, Republic of Korea

**Keywords:** core genes, database, identification, microbiome, species

## Abstract

With the continued evolution of DNA sequencing technologies, the role of genome sequence data has become more integral in the classification and identification of Bacteria and Archaea. Six years after introducing EzBioCloud, an integrated platform representing the taxonomic hierarchy of Bacteria and Archaea through quality-controlled 16S rRNA gene and genome sequences, we present an updated version, that further refines and expands its capabilities. The current update recognizes the growing need for accurate taxonomic information as defining a species increasingly relies on genome sequence comparisons. We also incorporated an advanced strategy for addressing underrepresented or less studied lineages, bolstering the comprehensiveness and accuracy of our database. Our rigorous quality control protocols remain, where whole-genome assemblies from the NCBI Assembly Database undergo stringent screening to remove low-quality sequence data. These are then passed through our enhanced identification bioinformatics pipeline which initiates a 16S rRNA gene similarity search and then calculates the average nucleotide identity (ANI). For genome sequences lacking a 16S rRNA sequence and without a closely related genomic representative for ANI calculation, we apply a different ANI approach using bacterial core genes for improved taxonomic placement (core gene ANI, cgANI). Because of the increase in genome sequences available in NCBI and our newly introduced cgANI method, EzBioCloud now encompasses a total of 109 835 species, of which 21 964 have validly published names. 47 896 are candidate species identified either through 16S rRNA sequence similarity (phylotypes) or through whole genome ANI (genomospecies), and the remaining 39 975 were positioned in the taxonomic tree by cgANI (species clusters). Our EzBioCloud database is accessible at https://www.ezbiocloud.net/db.

## Data availability

All data presented in this article can be found as supplementary files. The hierarchical taxonomy can be searched and browsed at through https://www.ezbiocloud.net/dbwww.ezbiocloudpro.app/en/databasewww.ezbiocloud.net/db.

## Introduction

Modern taxonomy of Bacteria and Archaea aims for an objective definition of species, essential for the purposes of identification and quantification. Since publishing our first EzBioCloud article [1], we have seen a dramatic increase in the number of genome sequences publicly available, from 62 362 in 2017, to 1 863 350 as of March 2024, a near 30-fold increase in 7 years. This surge has profoundly impacted our understanding and approaches to microbial taxonomy, especially in the context of real-world applications. One such area is in microbiome shotgun analysis, particularly in environmental and animal microbiome research. In these fields, many bacteria and archaea remain either uncultured or without valid name, presenting unique challenges for accurate species identification and quantification.

Traditional methods like PCR and sequencing of 16S rRNA genes have laid the groundwork for understanding bacterial and archaeal phylogeny. However, limitations arise when these methods are applied at the species level, especially in the case of nearly identical 16S rRNA gene sequences that do not guarantee species congruence [2]. While DNA–DNA hybridization offered a complementary approach, it has largely been supplanted by genome-based classifications. In this context, average nucleotide identity (ANI) and OrthoANI [3] have been instrumental in defining species boundaries, but their effectiveness diminishes for novel branches of the bacterial tree, where no close comparative sequences exist [4].

To address these challenges, we introduce core gene ANI (cgANI), a core gene method for initial taxonomic placement in the phylogenetic tree of bacteria. This method facilitates further analysis with ANI and is particularly valuable for genome sequences that do not have or have only a partial 16S rRNA sequence, where a complete 16S rRNA sequence is often necessary for accurate taxonomic assignment [5,6]. The integration of over a million quality-filtered genomes into databases like EzBioCloud has not only expanded our genomic resources but also necessitated improvements in metadata curation and taxonomic accuracy. EzBioCloud now offers a comprehensive, quality-controlled genome database of every type strains, with taxonomic identification at genus, species, and subspecies levels using a combination of gene-based searches and OrthoANI calculations. This database serves as an invaluable tool for a wide array of microbiological disciplines, from basic research to clinical applications, enhancing our capacity to navigate the ever-expanding landscape of microbial diversity.

## Methods

### Data collection and curation

We obtained 16S rRNA gene and genome sequences of type strains through two primary methods. Firstly, we sourced novel taxa primarily from papers published in the *International Journal of Systematic and Evolutionary Microbiology* (IJSEM). Secondly, we utilized the NCBI taxonomy database, along with information from the NCBI Assembly Database and the NCBI Nucleotide Database.

Subsequently, we conducted sequence identification and taxonomy verification to ensure consistency with the information reported in the literature. Following the International Code of Nomenclature of Prokaryotes (ICNP) [7], we assigned higher priority to a taxon when a sequence exhibited similarity beyond the species boundary with the taxon’s reference genome [8,9]. A reference genome is a fully sequenced and extensively annotated genome of a representative bacterial strain, serving as a standard for comparative genomics. For valid named species, the reference genome is usually that of the type strain. For those without a type strain, the highest quality genome available (N50, assembly depth) is selected as representative.

Next, we manually aligned the sequences based on the secondary structure of the 16S rRNA gene, while rigorously assessing sequence quality using the EzEditor program [10]. Finally, we employed neighbour-joining phylogenetic trees to validate taxonomy assignments and utilized ANI and the core gene tree with the up-to-date bacterial core gene (UBCG) set [11], which are a group of 92 single-copy core genes that are present in most bacteria, to confirm taxonomic groupings based on the manually aligned 16S rRNA gene sequences.

### cgANI

The cgANI is calculated using the UBCG set, which includes 92 core genes. The UBCG set was extracted from each genome by utilizing a pipeline implemented by Na *et al*. [11] with default parameters. The UBCG pairs were aligned with each other using the semi-global alignment algorithm supported by the Parasail package [12] in Python. The cgANI values are determined by averaging the identity values obtained from all UBCG pairs.

### Genome-based taxonomic identification

**Fig. 1.**
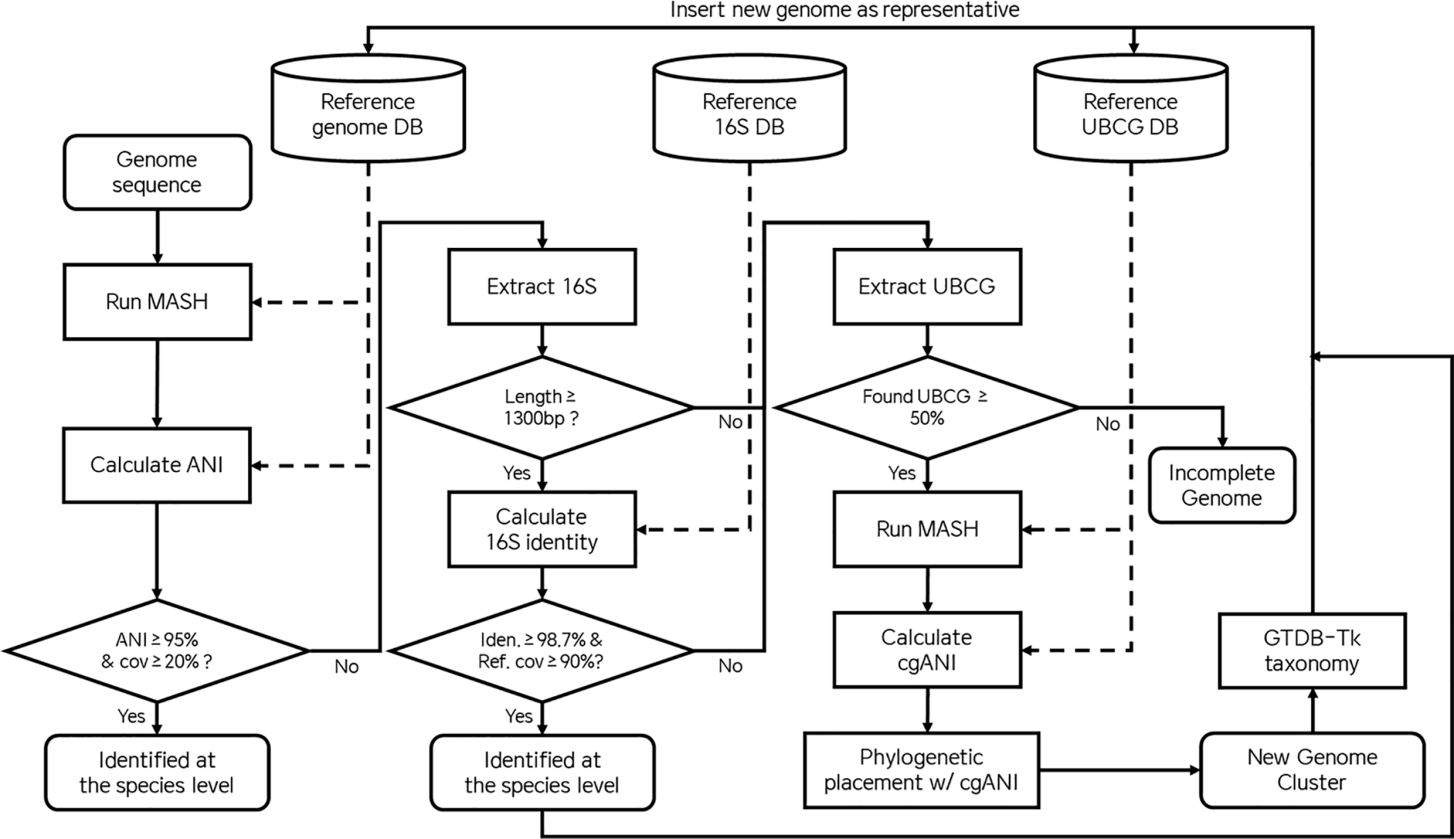
Workflow for classifying microbial genomes. It illustrates initial genome similarity assessment, species-level identification through ANI and 16S rRNA analysis, followed by phylogenetic placement using UBCG sequences. The process ends with taxonomic validation and database integration, improving future classification accuracy.

In the delineated taxonomic classification workflow as show in Fig. 1, microbial genomic sequences undergo a preliminary similarity assessment via MASH [13], which employs MinHash techniques to estimate genome-wide distances. Subsequent to this initial screening, the ANI is computed to quantify the similarities with reference genomes. A genome achieving an ANI of 95 % or more, with a minimum coverage of 20 %, is confidently classified at the species level [8,9]. Failing to meet these criteria, the procedure advances to extract and analyse the 16S rRNA gene, provided it surpasses the 1300 bp threshold. If the extracted 16S rRNA sequence demonstrates greater than 98.7 % identity and a reference genome’s coverage exceeds 90 %, it is identified at the species level. If both of the previous steps fail to identify the genome sequence, then we proceed to extract the UBCG sequences for cgANI-based phylogenetic placement. First, MASH and a database containing the UBCG sequences from the reference genomes are used to select the top 10 closest hits, followed by the calculation of cgANI as previously described. Using FastTree [14], we created a phylogenetic tree and manually curated the taxonomy using the lowest common ancestor approach. Finally, we validated the taxonomic placement using GTDB-tk [15], following their taxonomic nomenclature up to genus level. All new genome cluster representative sequences are given a placeholder name using the prefix MSSC (Microbiome Strain Sequence Cluster) followed by a numerical identifier. This process concludes with the incorporation of the genome sequence into our database as a representative sequence. Consequently, when another closely related genome requires classification, it will undergo initial identification using the ANI classification method, leveraging the newly added representative sequence for enhanced accuracy and efficiency.

### Metagenome profiling

In our metagenome profiling method, we start the process by assessing the presence of bacterial and archaeal species in each raw metagenomic sample. This assessment is performed using Kraken2 [16] coupled with a core gene database [17], which includes k-mers (k=35) derived from reference genomes in the EzBioCloud database. Subsequently, a list of potential candidate species is generated. Utilizing these species, we construct a custom bowtie2 database [18], which is based on the core gene sequences from the species identified in the previous step. The raw metagenomic sample is then aligned to this bowtie2 database with default values. The alignment output is processed using Samtools [19] to convert and sort the resulting bam files. We then determine the coverage of the mapped reads against the bam file using Bedtools [20]. To minimize false-positive identifications, an in-house script is deployed to ensure that reads are attributed to a specific species only if they meet a minimum coverage threshold of 25 % coverage across their core genes. Finally, the abundance of each species is quantified based on the total count of mapped reads. We normalize species abundance by factoring in the total length of all the core genes from the reference genomes.

## Results and discussion

### Taxonomy structure

The EzBioCloud database has undergone remarkable expansion in its hierarchical taxonomic system since 2017, showcasing the advancements in microbial genomics and taxonomy. Currently, the database comprises 340 phyla, 845 classes, 2524 orders, 6887 families, 28 808 genera, 109 835 species, and 470 subspecies. This significant growth from 2017, which included 207 phyla, 433 classes, 1019 orders, 2805 families, 11 446 genera, 61 700 species, and 387 subspecies, highlights the rapid evolution in the field of microbial taxonomy.

### Core gene-derived taxonomy

The EzBioCloud database has seen a substantial increase in its representative species, primarily due to the significant growth in the number of genomes analysed – from 62 362 in 2017 to 1 185 794 in 2024. This expansion in genomic data has been instrumental in enhancing species representation and diversity. A key factor in this growth has been the implementation of the cgANI method, which allowed for 39 975 cluster species to be placed in the taxonomic hierarchy. This method, serving as a complementary tool rather than a substitute for standards such as the 98.7 % similarity threshold for 16S rRNA gene sequencing or the 95 % ANI threshold, is particularly effective in identifying novel candidate species when traditional methods are insufficient. The cgANI method’s utility in establishing novel branches within phylogenetic trees and calculating identity between well-conserved gene pairs makes it applicable even for distantly related taxa, overcoming the limitations of ANI in cases where reference genomes show little overlap. Thus, cgANI not only facilitates the inclusion of a larger number of tentatively named species but also provides a robust framework for phylogenetic placement and whole-genome ANI analyses, enriching the EzBioCloud database with a nuanced understanding of microbial diversity.

### Identification of genome clusters in human microbiome analyses

**Fig. 2.**
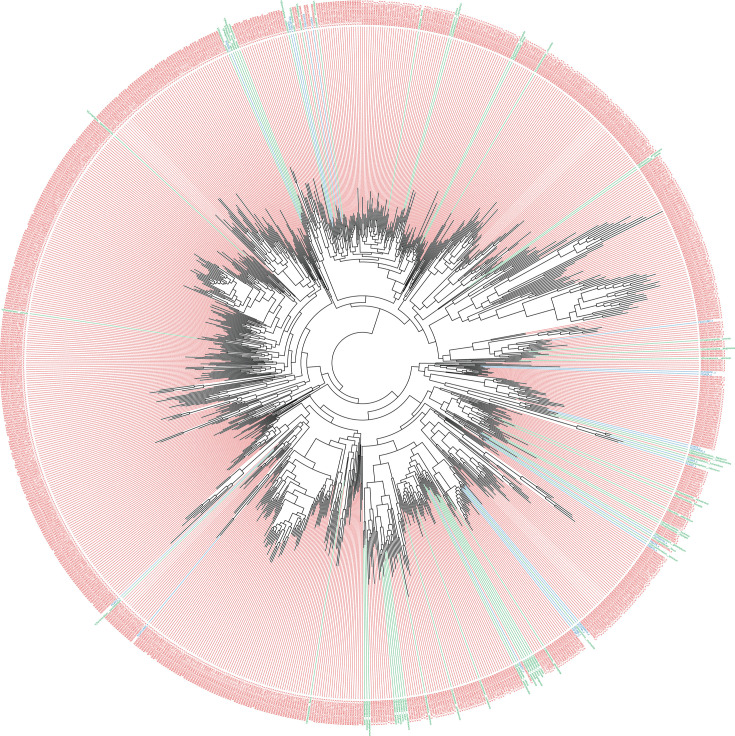
Taxonomy of the phylum *Verrucomicrobiota*, illustrating species represented by genome sequence with validly recognized names in green and genome cluster representatives in red, demonstrating the need and the effectiveness of the cgANI method in microbial taxonomy.

The phylum *Verrucomicrobiota* exemplifies the effectiveness of the cgANI method in microbial taxonomy. Within this phylum, there are 67 species with validly recognized names, alongside 1243 phylotypes and 856 genome cluster representatives (Fig. 2). The phylotype representatives were determined using traditional methods like 16S rRNA gene 98.7 % similarity or ANI 95 % genome similarity, as previously described [1]. In contrast, the 856 genome clusters represent species that were identified through the cgANI method, highlighting its utility in classifying species that traditional methods might not fully resolve. This approach, has led to the identification and categorization of numerous species within *Verrucomicrobiota*, showcasing the method’s strength in enhancing our comprehension of microbial diversity and the phylogenetic landscape.

In order to showcase this, we analysed 6882 microbiome samples from healthy individuals to search for *Verrucomicrobiota* and found that 2,000 samples contained validly named species, while 948 samples contained either a genomospecies or a genome cluster from that phylum (Table S1, available in the online version of this article). The genus *Akkermansia* was the most prevalent, comprising five validly named species, three genomospecies, and seven genome clusters (Table S2). In total, 36 species genome clusters were identified. To ensure the accuracy of the identifications, we only considered classifications where reads covered at least 25 % of the core genes, thereby eliminating potential spurious identifications (Table S3).

**Fig. 3.**
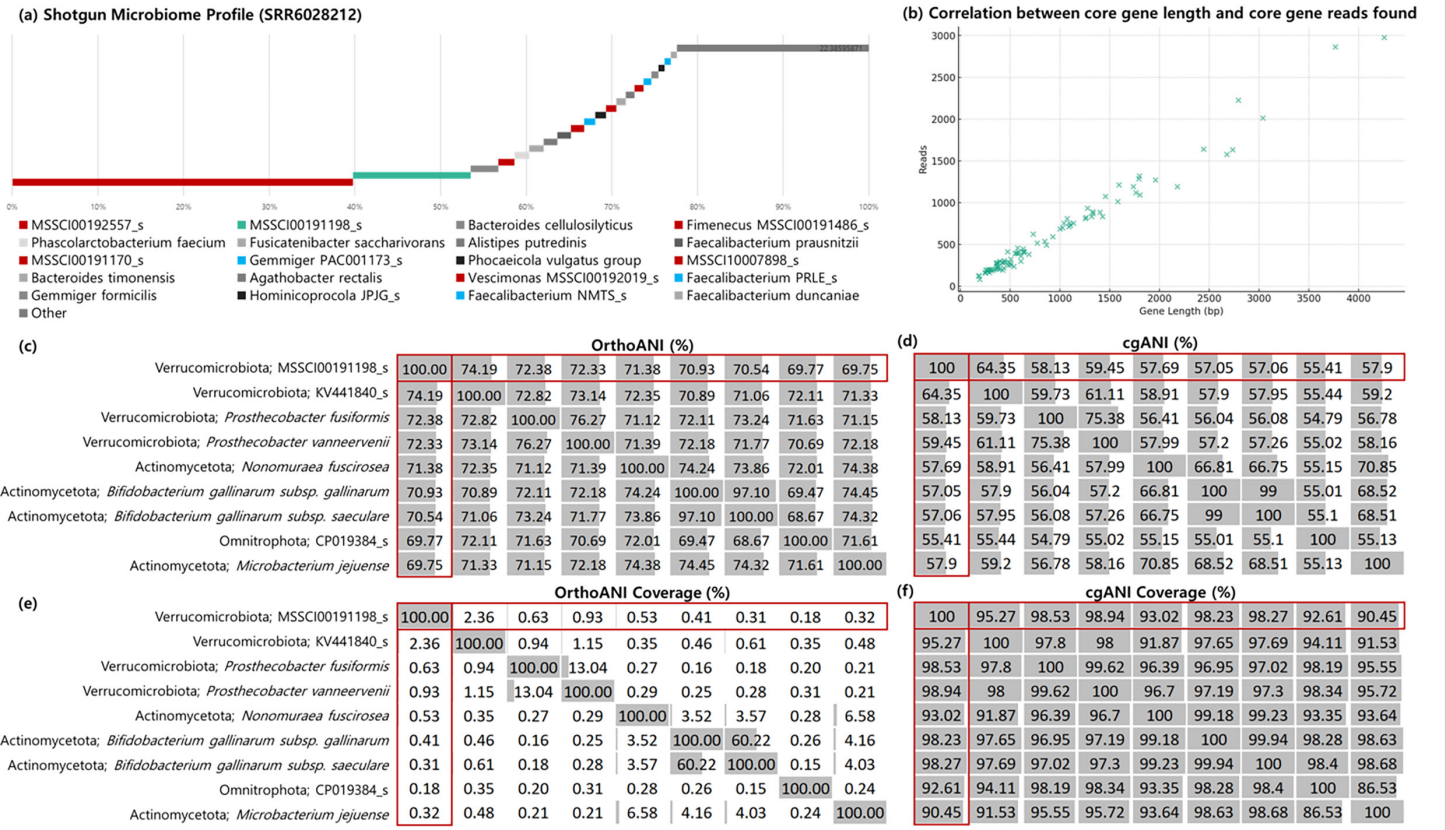
Microbiome profile analysis and genomic cluster identification. This figure presents a comprehensive analysis of a microbiome sample, highlighting the significance of genome cluster MSSCM00191198_s from the phylum *Verrucomicrobiota*. (**a**) Microbiome profile with MSSCM00191198_s as the top hit. (**b**) Correlation between gene length and the number of reads classified to MSSCM00191198_s, indicating precise quantification. (**c**) and (**d**) OrthoANI and cgANI matrices, respectively, comparing MSSCM00191198_s with its closest genome sequences, demonstrating the specificity of genomic similarities. (**e**) and (**f**) OrthoANI and cgANI coverage matrices, respectively, further detailing the extent of sequence overlap and coverage.

Fig. 3(a) illustrates one of these microbiome profiles, where it can be seen that the inclusion of genome clusters in databases is crucial. The top two species represented in the sample are genome clusters, with the second one belonging to the phylum *Verrucomicrobiota* (MSSCM00191198_s), which is highlighted in green. Notably, relying solely on validly named species in our database would impede correct identification of this genomic cluster (tentative species) in Fig. 3(a). To assess whether this identification is a true or a false positive, we compared each one of the core gene sequences against the number of reads classified to each of those genes. In Fig. 3(b) we can observe a near linear correlation between the length of each of the genes against the number of reads classified to them, demonstrating not only that all genes were found for that genome cluster, but the correlation also demonstrates accurate quantification.

Additionally, we explored the possibility of the genomic cluster, MSSCM00191198_s, which lacks closely related species with valid names or genomospecies, demonstrating the limitations of traditional taxonomy databases (Fig. 3(c)). The application of cgANI analysis significantly improves species delineation, surpassing orthoANI, where the ANI percentage frequently represents less than 1 % of the sequence overlap (Fig. 3(e)). This precision of cgANI in detecting genetic similarities can be seen in the matrix, where it demonstrates enhanced discrimination among clusters. Such evidence conclusively affirms the unique identity of the genomic clusters under consideration.

The employment of cgANI for the identification of genomic clusters, such as MSSCM00191198_s bridges the gap between rapid technological advances and conventional taxonomy. In the pursuit of scientific clarity and utility, there are numerous fields where the immediate identification and discrimination of bacterial strains take precedence over taxonomic labelling. This is especially important in the field of biomarker discovery, in clinical trials, and translational studies bridging animal models and human subjects, where the precise characterization of microbial entities can drive pivotal outcomes. Despite the dynamic and often constant change in the nature of microbial taxonomy, databases such as EzBioCloud remain dedicated to the unification and standardization of validly named species. To accommodate the exigencies of scientific research, the incorporation of genomic clusters and genomospecies serves as a necessary interim measure. This allows for the provisional classification of bacterial entities while the taxonomic framework catches up with the rapid pace of genome sequencing and discovery. This ensures that researchers have access to the most current and accurate data, underpinning continuous updates that reflect the evolving landscape of microbial taxonomy. Thus, while the need for rapid identification is important in various applications, the maintenance of a standardized taxonomic framework is essential, serving as the backbone for robust scientific research and its applications in medicine and beyond.

## supplementary material

10.1099/ijsem.0.006421 Uncited Table S1.

## Abbreviations

ANI: average nucleotide identity
cgANI: core gene average nucleotide identity
MSSC: microbiome strain sequence cluster
UBCG: up-to-date bacterial core genes
